# Pharmacological Modulation of Rate-Dependent Depression of the Spinal H-Reflex Predicts Therapeutic Efficacy against Painful Diabetic Neuropathy

**DOI:** 10.3390/diagnostics11020283

**Published:** 2021-02-11

**Authors:** Corinne A. Lee-Kubli, XiaJun Zhou, Corinne G. Jolivalt, Nigel A. Calcutt

**Affiliations:** Department of Pathology, University of California San Diego, 9500 Gilman Drive, La Jolla, CA 92093, USA; cleekubli@salk.edu (C.A.L.-K.); xiajunzhou76@163.com (X.Z.); cjolivalt@ucsd.edu (C.G.J.)

**Keywords:** diabetic neuropathy, painful neuropathy, spinal disinhibition, GABAergic, carbonic anhydrase, rate dependent depression, KCC2, GABA_B_ receptor, baclofen, acetazolamide

## Abstract

Impaired rate-dependent depression (RDD) of the spinal H-reflex occurs in diabetic rodents and a sub-set of patients with painful diabetic neuropathy. RDD is unaffected in animal models of painful neuropathy associated with peripheral pain mechanisms and diabetic patients with painless neuropathy, suggesting RDD could serve as a biomarker for individuals in whom spinal disinhibition contributes to painful neuropathy and help identify therapies that target impaired spinal inhibitory function. The spinal pharmacology of RDD was investigated in normal rats and rats after 4 and 8 weeks of streptozotocin-induced diabetes. In normal rats, dependence of RDD on spinal GABAergic inhibitory function encompassed both GABA_A_ and GABA_B_ receptor sub-types. The time-dependent emergence of impaired RDD in diabetic rats was preceded by depletion of potassium-chloride co-transporter 2 (KCC2) protein in the dorsal, but not ventral, spinal cord and by dysfunction of GABA_A_ receptor-mediated inhibition. GABA_B_ receptor-mediated spinal inhibition remained functional and initially compensated for loss of GABA_A_ receptor-mediated inhibition. Administration of the GABA_B_ receptor agonist baclofen restored RDD and alleviated indices of neuropathic pain in diabetic rats, as did spinal delivery of the carbonic anhydrase inhibitor acetazolamide. Pharmacological manipulation of RDD can be used to identify potential therapies that act against neuropathic pain arising from spinal disinhibition.

## 1. Introduction

One of the challenges to the effective treatment of neuropathic pain is that current frontline analgesics have unpredictable efficacy and an unclear mechanism(s) of action. Animal studies suggest that pain states may be peripherally or centrally mediated [1,2] and pain may derive from diverse mechanisms, even within a single disease class. Identifying the pain generator site(s) in individual patients is therefore an important step in selecting appropriate therapies. Recent clinical trials support this approach, as drugs impeding sodium channel function were found to be effective specifically in patients with an irritable nociceptor phenotype that is suggestive of peripherally driven pain [3,4], while microneurography of peripheral nerves has been used to select subjects with spontaneous activity for trials of a calcium channel blockers [5]. Conversely, we have demonstrated that impaired rate-dependent depression (RDD) of the spinal Hoffman (H-) reflex occurs in models of painful neuropathy associated with spinal disinhibition, such as diabetes, while it is normal in a model of paclitaxel-induced neuropathic pain, which is thought to result from peripheral dysfunction [6]. RDD is readily evaluated in both animals [7] and humans [8] and we recently confirmed that loss of RDD in diabetic rodents translates to humans, as RDD was significantly impaired in patients with painful diabetic neuropathy while it remained normal in patients with painless diabetic neuropathy [9]. Furthermore, RDD appeared to be most impaired in a sub-population of patients that receive therapeutic benefit from duloxetine, a serotonergic agonist that may act by restoring spinal inhibitory function [10]. This underlines the potential clinical utility of RDD as a diagnostic tool for identifying patients in whom spinal disinhibition contributes to chronic pain.

The phenomenon of RDD has been known for over half a century [8]. Peripheral nerve stimulation causes direct orthodromic activation of motor axons that drives contraction of the corresponding muscle, resulting in a short-latency response (M-wave) detected by electromyogram (EMG). The longer-latency H-reflex arises from stimulation of sensory axons that synapse in the spinal cord, ultimately resulting in depolarization of ventral horn motor neurons and a second muscular contraction seen as a longer-latency response by EMG [8,11]. The H-reflex can undergo different types of modulation in response to preceding stimulation, including a low-frequency depression within 0.2–2.0 s of the preceding stimulation [12]. This low-frequency depression has variably been referred to as RDD, frequency-dependent depression, or paired-pulse depression [12,13,14,15]. To date, RDD of the H-reflex has been most commonly used to assess spinal inhibitory function in models of spinal cord injury and stroke with concomitant spasticity and rigidity [16,17,18], and recovery of inhibitory function following therapeutic interventions [19].

In normal rats, RDD depends upon the inhibitory function of spinal GABA_A_ receptors, as RDD is blocked by the GABA_A_ receptor antagonist bicuculline [7]. Loss of RDD in diabetic rats is not associated with reduced expression of spinal GABA_A_ receptors [7] or diminished spinal GABA release—indeed both basal and stimulus-evoked release of GABA is exaggerated in these animals [20]. Loss of RDD in diabetic rats is associated with spinal disinhibition resulting from reduced dorsal spinal expression of the potassium-chloride co-transporter (KCC2). Under normal conditions, KCC2 expression by post-synaptic neurons of the spinal cord maintains low intra-neuronal chloride, such that opening the GABA_A_ receptor leads to an influx of chloride and membrane hyperpolarization [21]. However, when spinal KCC2 expression and/or activity is reduced, intra-neuronal chloride accumulates, so that opening of GABA_A_ receptors results in chloride efflux and subsequent depolarization of the post-synaptic membrane [22]. Loss of RDD in diabetic rats can be replicated in normal rats by spinal delivery of the KCC2 inhibitor DIOA or of the neurotrophic factor BDNF, which reduces expression of KCC2 [6,7]. Inhibiting GABA_A_ receptors restores RDD in normal rats treated with DIOA, as well as in diabetic and BDNF-treated rats, suggesting the presence of alternative spinal inhibitory systems that can also contribute to RDD.

Because loss of RDD may serve as a biomarker for spinal disinhibition in neuropathic pain states [9], the contribution of alternative inhibitory systems to RDD may allow development of novel therapeutic strategies against spinally driven pain. We have therefore extended investigation of the spinal pharmacology of RDD in normal and STZ-diabetic rats to identify alternative contributors to RDD. Major findings are that early loss of spinal KCC2 protein and GABA_A_ inhibitory function in diabetic rats does not initially result in loss of RDD due to the continued inhibitory function of GABA_B_ receptors. Later onset of RDD impairment is linked to a decline in GABA_B_ receptor inhibitory function. We additionally demonstrate that a carbonic anhydrase inhibitor, acetazolamide, restores RDD by enhancing GABA_A_ receptor function and also alleviates tactile allodynia in diabetic rats.

## 2. Material and Methods

Animals and induction of diabetes: Studies were performed using adult female Sprague–Dawley rats (Envigo, San Diego, CA, USA). Animals were housed 2 per cage with free access to food (5001 diet, Purina, Lab Supply, Fort Worth, TX, USA) and tap water. All studies were carried out according to protocols approved by the Institutional Animal Care and Use Committee of the University of California, San Diego in a vivarium approved by the American Association for the Accreditation of Laboratory Animal Care. Type 1 diabetes was induced in 13-week old female Sprague-Dawley rats by a single i.p. injection of streptozotocin (STZ, Sigma, St. Louis, MO, USA) at 50 mg/kg freshly dissolved in 0.9% sterile saline following an overnight fast. Only STZ-injected rats with non-fasting blood glucose levels of ≥15 mmol/L as determined using a strip-operated reflectance meter (OneTouch Ultra, LifeScan, Inc., Milpitas, CA, USA), in blood samples obtained by tail prick both four days after STZ injection and at the conclusion of the study were considered diabetic. Rats were studied 4 or 8 weeks after the onset of diabetes.

Drugs: The GABA_A_ antagonist bicuculline (0.6 µg; TCI America, Portland, OR, USA) and the GABA_B_ agonist baclofen (0.0003–0.1 µg; Sigma, St. Louis, MO, USA) were dissolved in sterile saline. The selective KCC2 blocker DIOA (3 µg; Alexis Biochemicals, San Diego, CA, USA) was dissolved in saline + 10% DMSO. The GABA_B_ antagonist phaclofen (10 µg; Alexis Biochemicals, San Diego, CA, USA) was dissolved in saline + 20% 0.1 N HCl, pH adjusted to 7.0. The carbonic anhydrase inhibitor acetazelomide (22.5 µg; Sigma, St. Louis, MO, USA) was dissolved in phosphate-buffered saline (PBS). Vehicles were matched by composition and pH for each drug. Drug formulations and doses were selected on the basis of previous studies [7,10,23]. Drug or vehicle was delivered direct to the spinal cord via an indwelling intrathecal (IT) catheter that was implanted 3–7 days prior to drug delivery, as described in detail elsewhere [24]. Bicuculline, baclofen, phaclofen, and acetazolamide were injected in a volume of 10 μL. Where two drugs were co-administered, a volume of 5 μL per drug was used. Drug administration was followed by 10 μL of saline to flush the catheter.

Rate-dependent depression: The H-reflex was recorded under isoflurane anesthesia. One hind limb of the rat was secured and a needle electrode (Grass Technologies, West Warwick, RI, USA) inserted at the ankle for stimulation of the adjacent tibial nerve. Two recording electrodes were inserted into interosseous muscles of the ipsilateral hind paw. Stimulus generation and recording of M- and H-waves from the resulting EMG were performed using a Powerlab 4/30 connected to a computer running Scope software (AD Instruments, Colorado Springs, CO, USA). The tibial nerve was stimulated using bursts of 5 × 200 μs duration square waves with 40 μs intervals between each square wave. The resulting M- and H-waves in response to each burst were recorded. For measurements of RDD curves, bursts were repeated at 0.2 to 5 Hz frequencies. For drug studies, bursts were repeated at a 1 Hz stimulation frequency because this frequency is associated with an approximately 40% decrease in the amplitude of the H-wave in normal rats, allowing for detection of changes in response to drug administration [6]. The stimulation intensity was increased by 0.125 V increments until the intensity that gave rise to H_max_ (defined as the maximum amplitude of the H-wave) was found. RDD was calculated as the percentage change in the amplitude of the H-wave evoked by the second stimulation (H2) compared to the H-wave amplitude evoked by the first (H1). Importantly, the M-wave did not decrease during stimulation at any frequency in any of the groups tested, indicating that RDD of the H-reflex does not involve fatigue of motor units or a shift in electrode placement. Mean % change in M-wave amplitude across groups ranged from −3.74 to 3.78% with standard deviations ranging from 2.89 to 8.61.

Tactile withdrawal threshold: The 50% paw withdrawal threshold (50% PWT) to a series of calibrated von Frey filaments (Kom Kare, Middletown, OH, USA) was assessed in the center of both hindpaws at baseline and at regular intervals after drug administration using the up-down method [25,26]. Values from the two hindpaws were averaged at each time point. Percent maximum potential effect (% MPE) was calculated as (PWT-baseline PWT)/(15-baseline PWT) * 100. This assumes that the maximal drug effect would be to increase the PWT to its maximum measurable output, 15 g. Area under the curve (AUC) was calculated using the trapezoidal rule.

Western Blotting: Spinal cords were obtained by hydraulic extrusion after decapitation of anaesthetized rats. The lumbar enlargement was dissected into dorsal and ventral portions and collected into ice-cold homogenization buffer (50 mM Tris–HCl, pH 7.4, 150 mM NaCl, 1 mM EDTA, 0.5% Triton X, protease inhibitor cocktail) and homogenized before centrifugation at 14,000× *g*. Protein (7.5–15 μg) was prepared by incubating in 4× NuPAGE sample buffer (Invitrogen, Carlsbad, CA, USA) for 30 min at 37 °C before being separated on 4–12% SDS–PAGE Bis–Tris gels (Novex, Invitrogen, Carlsbad, CA, USA) and immunoblotted on Amersham Hybond C-Extra nitrocellulose (GE Healthcare, Buckinghamshire, UK). Membranes were blocked with 5% bovine serum albumin (BSA; Sigma, St. Louis, MO, USA) before being incubated with anti-KCC2 (1:1000, Millipore, Temecula, CA, USA) or anti-actin antibodies (1:2000, Sigma, St. Louis, MO, USA) in 3% BSA. Membranes were then incubated with HRP-conjugated anti-rabbit and anti-mouse antibodies (1:10,000; Santa Cruz Biotechnology, Santa Cruz, CA, USA) followed by ECL Plus Western Blotting Detection System (GE Healthcare, Buckinghamshire, UK). Proteins were visualized on Amersham Hyperfilm ECL (GE Healthcare, Buckinghamshire, UK), scanned into a computer and quantified using QuantityOne software (BioRad, Hercules, CA, USA).

Statistical Analysis: Between-group statistical analyses were performed with GraphPad Prism (v 5.0; La Jolla, CA, USA) using either unpaired two-tailed t-test, one-way ANOVA followed by Tukey’s or Dunnett’s post-hoc tests for multiple comparisons, or two-way ANOVA followed by Bonferroni’s post-hoc test as indicated. Data are reported as group mean ± SEM. For experiments requiring multiple sequential injections of different drugs, baseline and first injection groups were large in order to allow subsequent separation into experimental groups that received a different second injection.

## 3. Results

### 3.1. Spinal Pharmacology of RDD in Naïve Rats

**Figure 1:**
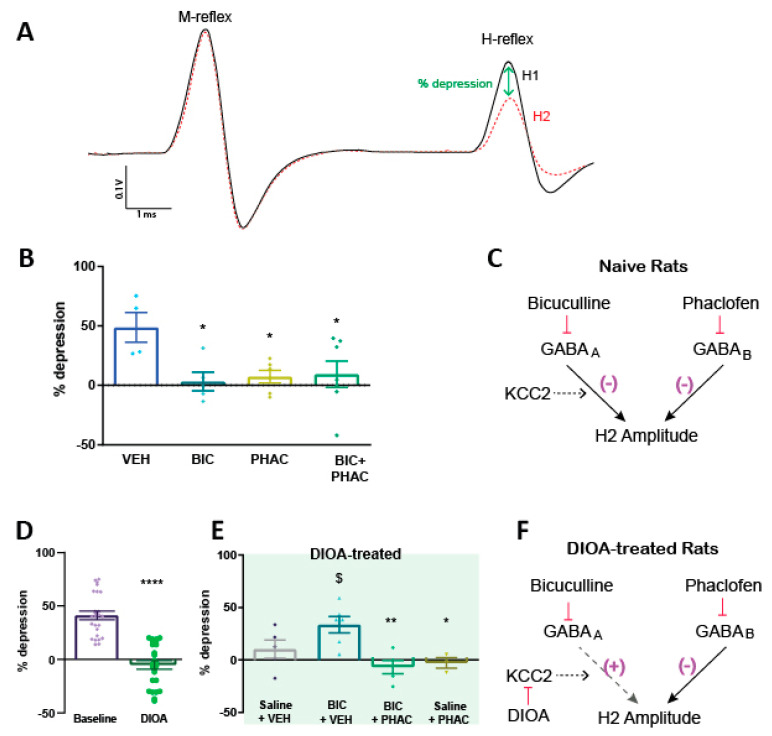
Pharmacology of RDD in 16-week-old female Sprague-Dawley rats. (**A**) Representative M- and H-reflex traces from naïve rats. % depression is calculated as the change in amplitude of the second recorded H-reflex (H2) compared to the first in the train (H1). (**B**) % depression of the H-reflex in response to 1 Hz stimulation frequency in 16-week old rats at baseline and 5 min after administration of vehicle (VEH), phaclofen (10 μg; PHAC), bicuculline (0.6 μg; BIC) or combined bicuculline and phaclofen (BIC + PHAC). * *p* < 0.05, ** *p* < 0.01 compared to vehicle (baseline) by one-way ANOVA followed by Dunnett’s post-hoc test. N = 4–6 per group. (**C**) Schematic indicating hypothesized contribution of GABA_A_ and GABA_B_ receptors to depression of the H2 amplitude. (**D**) RDD in response to 1 Hz stimulation frequency in 16-week old rats (n = 25) before (Baseline; purple circles) and 5 min after (3 μg; green open circles) IT administration of DIOA. **** *p* < 0.001 by paired two-tailed *t*-test. (**E**) One minute later (6 min after DIOA administration), DIOA-treated animals received IT injections of either bicuculline (0.6 μg) and vehicle (BIC + VEH; upward teal triangles), bicuculline (0.6 μg) and phaclofen (10 μg; BIC + PHAC; green diamonds), saline and phaclofen (10 μg; Saline + PHAC; downward olive triangles), or saline and vehicle (Saline + VEH; black circles) and RDD were assessed 10 min later (16 min after DIOA administration). ** *p* < 0.01, * *p* < 0.05 compared to BIC +VEH (upward teal triangles), $ *p* < 0.05 compared to within subject DIOA-treatment baseline by two-way ANOVA followed by Sidak’s multiple comparisons test. n = 3–6 per group. (**F**) Representative schematic of effect of DIOA on GABA_A_ receptor-mediated contribution to H-reflex depression.

We have previously shown that RDD depends upon spinal GABA_A_ receptor-mediated inhibition [7]. As GABA_A_ and GABA_B_ receptors are evenly distributed throughout the spinal gray matter [27] and may play similar roles in modulation of pain [28], we extended the pharmacological characterization of RDD to determine the contribution of GABA_B_-receptor mediated inhibition. Both bicuculline (GABA_A_ receptor antagonist) and phaclofen (GABA_B_ receptor antagonist) produced a significant (*p* < 0.05 compared to vehicle) impairment of RDD in naïve rats within 5 min of administration, whereas co-administration did not produce any notable additive effect (Figure 1A), suggesting that GABA_A_ and GABA_B_-mediated inhibition are both part of the inhibitory circuitry contributing to RDD (Figure 1B). The apparent contribution of GABA_B_ receptors to RDD prompted us to evaluate whether they are involved in the previously observed bicuculline-mediated restoration of RDD in naïve rats pre-treated with the KCC2 blocker DIOA [7]. Administration of DIOA produced a pronounced impairment of RDD within 5 min of administration (Figure 1B). Subsequent delivery of the bicuculline + vehicle, significantly (*p* < 0.001) restored RDD 10 min later. This effect was not due to washout of DIOA as neither saline + vehicle nor saline + phaclofen had an effect on RDD in DIOA pre-treated rats. Co-administration of phaclofen with bicuculline prevented restoration of RDD (Figure 1B; *p* < 0.05 compared to bicuculline + vehicle), indicating that the restoration of RDD by bicuculline depends upon ongoing GABA_B_ receptor-mediated inhibition.

### 3.2. Time Course of RDD and Spinal KCC2 Protein Expression in Diabetic Rats

**Figure 2:**
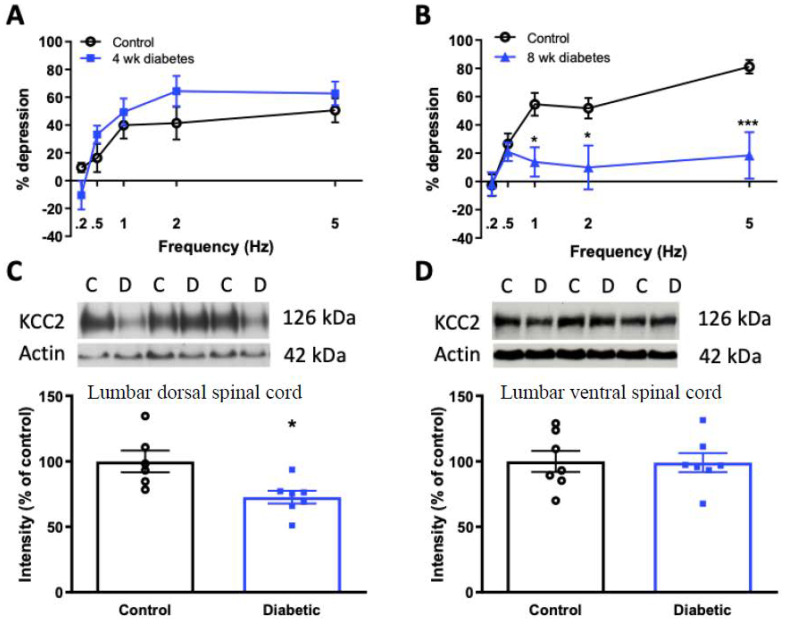
RDD and KCC2 expression in diabetic rats. % depression of the H-reflex over consecutive stimulation at 0.2–5 Hz frequencies in (**A**) 4-week diabetic rats (blue squares) and (**B**) 8-week diabetic rats (blue triangles), compared to age-matched non-diabetic controls (closed black circles). * *p* < 0.05, *** *p* < 0.001 by two-way ANOVA followed by Bonferonni’s post-hoc test. n = 11–3 per group. (**C**) Upper panel: KCC2 and actin protein in the lumbar dorsal spinal cord of control (**C**) and 4-week diabetic (**D**) rats. Lower panel: KCC2 intensity by Western blot normalized to actin loading control. (**D**) Upper panel: KCC2 and actin protein in the lumbar ventral spinal cord of control (**C**) and 4-week diabetic (**D**) rats. Lower panel: KCC2 intensity by Western blot normalized to actin loading control. Data are presented as group mean ± SEM. * *p* < 0.05 by unpaired two-tailed t-test. n = 7 per group.

All STZ–injected rats entered into the diabetic group exhibited blood glucose levels >20 mmol/L 4 days after injection of STZ and at sacrifice. After 4 weeks of diabetes, RDD was comparable to that of age-matched non-diabetic rats across all frequencies tested (Figure 2A). In contrast, but in agreement with our prior studies [6,7], RDD was significantly impaired across 1–5 Hz frequencies after 8 weeks of diabetes compared to age-matched non-diabetic rats (Figure 2B). We have previously shown that KCC2 protein is reduced in the dorsal lumbar spinal cord after 8 weeks of diabetes [6], and therefore evaluated spinal KCC2 expression in 4-week diabetic rats. Densitometric quantification of KCC2 protein in lumbar spinal cord from 4-week diabetic rats indicated a significant decrease in dorsal (Figure 2C), but not ventral (Figure 2D), spinal cord compared to age-matched naïve rats. Thus, reduced dorsal horn KCC2 protein expression precedes onset of the loss of RDD.

### 3.3. Spinal Pharmacology of RDD after 4 Weeks of Diabetes

**Figure 3:**
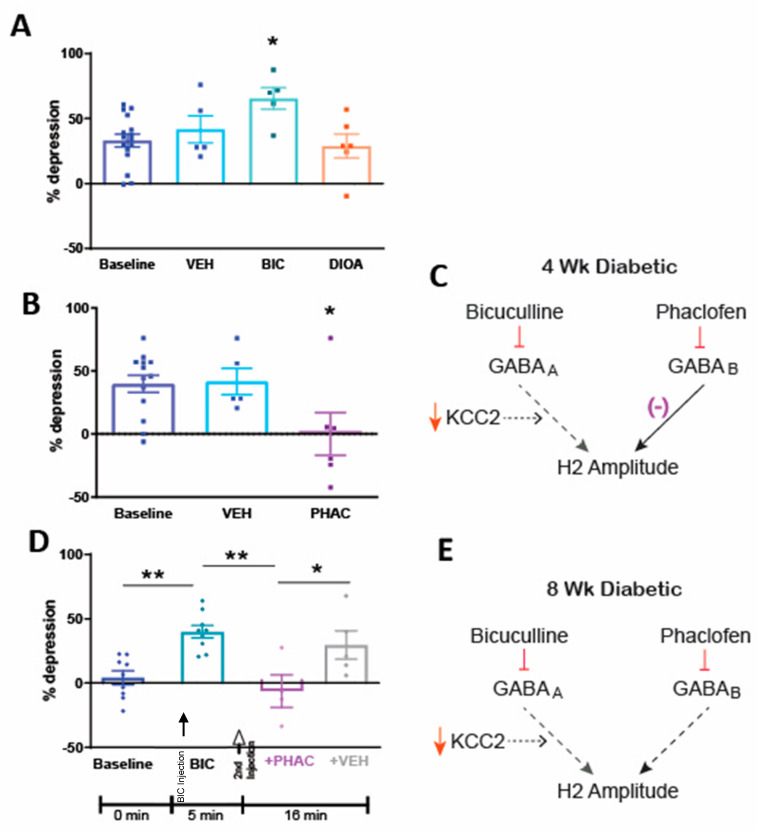
GABA_A_ and GABA_B_ receptor-mediated effects on RDD in 4- and 8-week diabetic rats. (**A**) % depression of the H-reflex in response to 1 Hz stimulation frequency in 4-week diabetic rats at baseline and 10 min after administration of vehicle, bicuculline (0.6 μg) or DIOA (3 μg). * *p* < 0.05 by one-way ANOVA followed by Dunnett’s post-hoc test. n = 5–16 per group. (**B**) % depression of the H-reflex in 4-week diabetic rats at baseline and 10 min after administration of vehicle or phaclofen (10 μg). * *p* < 0.05 by one-way ANOVA followed by Dunnett’s post-hoc test. n = 6–11 per group. (**C**) Schematic summarizing hypothesized contribution of GABA_A_ and GABA_B_ receptors to RDD of the H-reflex after 4 weeks of diabetes. (**D**) % depression of the H-reflex in response to 1 Hz stimulation frequency was evaluated in 8-week diabetic rats at baseline (blue diamonds) and 5 min after administration of bicuculline (BIC; 0.6 μg, teal diamonds). One minute later (6 min after bicuculline administration), either phaclofen (10 µg, + PHAC, purple diamonds) or vehicle (+ VEH, grey diamonds) were administered IT and RDD was assessed 10 min later. (**E**) Schematic summarizing hypothesized contribution of GABA_A_ and GABA_B_ receptors to RDD of the H-reflex after 8 weeks of diabetes. Data are presented as group mean ± SEM. ** *p* < 0.01, * *p* < 0.05 by one-way ANOVA followed by Tukey’s post-hoc test. n = 4–9 per group.

To resolve the apparent contradiction between reduced spinal KCC2 protein and normal RDD in 4-week diabetic rats, we evaluated the effect of bicuculline and DIOA, two drugs that impair RDD in normal rats [7]. Contrary to our findings in normal rats, DIOA had no effect on RDD and bicuculline significantly (*p* < 0.05) increased RDD compared to baseline (Figure 3A). In contrast, the GABA_B_ antagonist phaclofen significantly (*p* < 0.05) impaired RDD in 4-week diabetic rats 10 min after administration (Figure 3B). Taken together, these results suggest that the maintenance of normal RDD after 4 weeks of diabetes is mediated by functional GABA_B_ receptors, despite impaired GABA_A_ receptor-mediated inhibitory function (Figure 3C).

### 3.4. Spinal Pharmacology of RDD after 8 Weeks of Diabetes

RDD is impaired in 8-week diabetic rats and is restored to normal within 5 min of spinal bicuculline administration (Figure 3D and [13]). This finding is notable because, even in rats with inverted GABA_A_ receptor function due to reduced spinal KCC2 expression, functional blockade of GABA_A_ receptors would not be expected to enhance spinal inhibition, as GABA_A_ receptor-mediated inhibition would still be absent. It is possible that because GABA_A_ receptors in 8-week diabetic rats are no longer inhibitory due to the inverted chloride potential, blocking them unmasks the participation of another inhibitory system. To determine whether this restoration of RDD was due to the involvement of spinal GABA_B_ receptors, we delivered the GABA_B_ receptor antagonist phaclofen to the spinal cord 5 min after the administration of bicuculline. Phaclofen, but not vehicle, reversed the restoration of RDD by bicuculline (Figure 3D). The administration of phaclofen alone had no effect on RDD in 8-week diabetic rats (% depression at baseline = 1.4 ± 3.7 and 5 min after phaclofen = 3.8 ± 12.9). This suggests that RDD is impaired in rats after 8 weeks of diabetes due to failure of the inhibitory GABA_A_ receptor system and reduced inhibition through the GABA_B_ receptor system (Figure 3E).

### 3.5. Relevance of GABA_B_ Receptor-Mediated Inhibition to Neuropathic Pain in Rats

**Figure 4:**
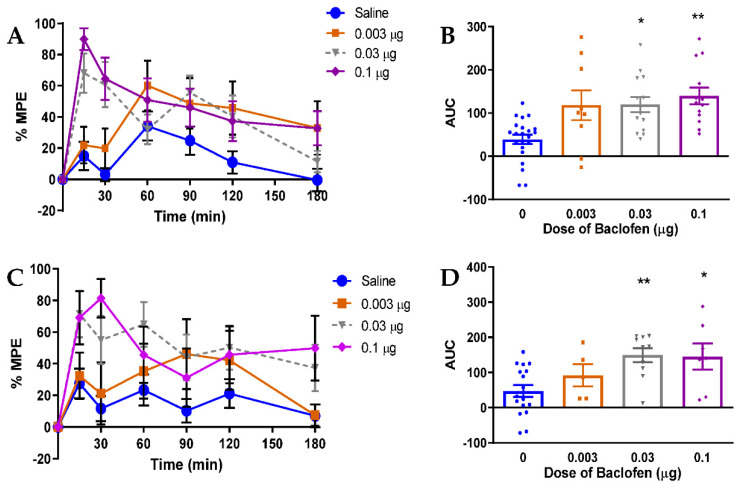
Effect of baclofen on tactile allodynia in 4- and 8-week diabetic rats. (**A**) % MPE of tactile allodynia over the 3 h following IT baclofen administration in 4-week diabetic rats. (**B**) Tactile allodynia dose-response AUC in 4-week diabetic rats following IT baclofen (0.003–0.1 µg) injection. (**C**) % MPE of tactile allodynia over the 3 h following IT baclofen administration in 8-week diabetic rats. (**D**) Tactile allodynia dose-response AUC in 8-week diabetic rats following IT baclofen (0.003–0.1 µg) injection. Tactile withdrawal threshold was assessed at 0, 15, 30, 60, 90, 120, and 180 min after injection. AUC was calculated using the trapezoidal method. * *p* < 0.05, ** *p* < 0.01 compared to saline (‘0′) by one-way ANOVA followed by Dunnett’s post-hoc test. n = 5–15/group.

The findings described above suggest that progressive failure of GABA_B_ receptor inhibitory function contributes to the onset of spinal disinhibition, as indicated by loss of RDD, in diabetic rats. We therefore investigated the therapeutic potential of spinal GABA_B_ receptor modulation by evaluating the efficacy of intrathecal administration of the GABA_B_ receptor agonist baclofen against indices of painful diabetic neuropathy in rats after 4 and 8 weeks of diabetes. Administration of baclofen produced dose-dependent alleviation of tactile allodynia in both 4- (Figure 4A,B) and 8-week (Figure 4C,D) diabetic rats. Doses greater than 0.10 μg were not used to due to the development of motor impairment.

### 3.6. Acetazolamide Reverses RDD Deficits and Alleviates Tactile Allodynia in Diabetic Rats

**Figure 5:**
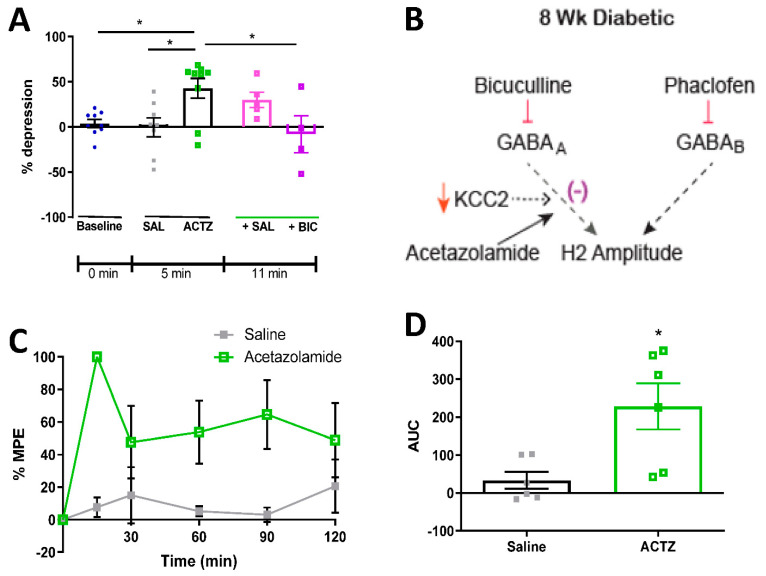
Effect of acetazolamide on RDD and tactile allodynia in diabetic rats. (**A**) % depression of the H-reflex in response to 1 Hz stimulation frequency in 8-week diabetic rats at baseline, 5 min after the administration of saline (SAL) or acetazolamide (ACTZ; 22.5 μg IT). One minute later (6 min after acetazolamide), saline (+Sal) or bicuculline (0.6 µg; + BIC) was administered IT and RDD was assessed 5 min later. * *p* < 0.05 compared to ACTZ by one-way ANOVA followed by Tukey’s post-hoc test. n = 4–9 per group. (**B**) Schematic summarizing of how acetazolamide restores RDD through enhancing GABA_A_ receptor-mediated inhibitory function. (**C**) Tactile response threshold in 8-week diabetic rats assessed at regular intervals after IT administration of acetazolamide (22.5 μg) expressed as % MPE relative to baseline values. (**D**) AUC of tactile withdrawal thresholds in 8-week diabetic rats after administration of saline or acetazolamide (22.5 μg). Data are presented as mean ± SEM. * *p* < 0.05 compared to saline by unpaired two-tailed t-test. N = 5–7 per group.

Allodynia arising from spinal chloride dysregulation and subsequent loss of GABA inhibitory function, as occurs when KCC2 expression or activity is impaired, can be corrected by the carbonic anhydrase inhibitor acetazolamide [23,29,30]. We therefore evaluated efficacy of acetazolamide against both RDD and tactile allodynia in STZ-diabetic rats. Acetazolamide (22.5 μg, IT) reversed RDD deficits within 5 min of administration to 8-week STZ-diabetic rats (Figure 5A). This was due to restoration of GABA_A_ receptor-mediated inhibition, as subsequent administration of bicuculline reinstated RDD impairment within 5 min of administration in acetazolamide pre-treated rats (Figure 5A). As previously reported [31], this suggests that acetazolamide acts by restoring GABA_A_ receptor-mediated inhibitory function (Figure 5B). Acetazolamide (22.5 μg, IT) also alleviated tactile allodynia in rats after 8 weeks of diabetes (Figure 5C,D), with peak efficacy occurring 15 min after administration and duration of effect of at least 2 h.

## 4. Discussion

In patients with equivalent neuropathy, loss of RDD identifies patients with painful versus painless diabetic neuropathy [9] and may represent a biomarker for the contribution of spinal disinhibition to neuropathic pain [6,32]. Understanding the pharmacology of both normal and impaired RDD is therefore of value for the identification of novel therapies to treating pain arising from spinal disinhibition. Our data demonstrates that both GABA_A_ and GABA_B_ receptor-mediated inhibition contributes to normal RDD and that the progression from normal to impaired RDD during diabetes is dependent on the relative contributions of both receptor sub-types. Therapies that restored RDD by manipulating spinal GABAergic pharmacology also corrected behavioral manifestations of neuropathic pain, indicating that restoration of RDD is a relevant predictor of behavioral outcome.

The attenuation of RDD in normal adult rats by bicuculline or phaclofen indicates that RDD depends upon concurrent GABA_A_ and GABA_B_ receptor-mediated inhibition. This is consistent with findings in cats which showed that paired-pulse depression of monosynaptic reflexes arises from both GABA_A_ and GABA_B_ receptor-mediated homosynaptic depression of the monosynaptic reflex arc [12,33]. The contribution of GABA_A_ receptor-mediated inhibition to RDD is dependent on spinal KCC2 [7,16]. When KCC2 function is compromised pharmacologically by DIOA, the resulting RDD impairments are reversed by administration of bicuculline in a GABA_B_ receptor-dependent fashion, suggesting an overlap between the inhibitory contributions of GABA_A_ and GABA_B_ receptors to RDD.

Our previous studies implicated depletion of spinal KCC2 protein in the mechanism by which diabetes induces pain-associated behaviors and RDD deficits [6,7]. KCC2 is predominantly expressed at inhibitory synapses in both the dorsal and ventral horn of the spinal cord [34] and an association between depletion of spinal KCC2 protein and loss of RDD has been found in animal models of spinal cord injury, STZ-induced diabetes, stroke, acute intrathecal administration of BDNF and genetic reduction of KCC2 protein expression [6,7,16,18]. It was therefore puzzling that RDD was normal after 4 weeks of diabetes, despite reduced spinal KCC2 protein levels. This could arise from disassociation of KCC2 protein levels and pump activity, as KCC2 activity can be post-translationally regulated [35,36,37], and active KCC2 in the cell membrane is more important than total KCC2 protein levels [38]. In order to address this discrepancy, GABA_A_ receptor function was evaluated in 4-week diabetic rats. Neither KCC2 inhibition (DIOA) nor GABA_A_ receptor antagonism (bicuculline) impaired RDD in 4-week diabetic rats, while bicuculline significantly increased RDD. This finding is similar to data from interferon-γ-treated rats and rats with spinal contusion injury in which spinal dorsal horn neurons have normal paired-pulse depression that is no longer impaired and can even be enhanced by bicuculline [39,40]. Likewise, we and others have also reported that bicuculline alleviates pain in rats with reduced spinal KCC2 expression, suggesting that bicuculline can dampen aberrant sensory function when the chloride reversal potential is presumably abnormal [7,41]. Our data suggest that, consistent with spinal KCC2 protein depletion at this duration of diabetes, GABA_A_ receptor function is already excitatory in 4-week diabetic rats, and that removing the influence of excitatory GABA_A_ receptors with bicuculline allows for increased inhibition via an alternative inhibitory system.

The suppression of RDD by phaclofen in 4-week diabetic rats indicates the presence of ongoing GABA_B_ receptor-mediated inhibitory function. This is consistent with participation of GABA_B_ receptors in RDD of normal rats, as discussed above, and suggests that the presence of a functional GABA_B_ receptor-mediated spinal inhibitory system masks GABA_A_ receptor-mediated excitation derived from reduced spinal KCC2 expression. However, the ability of GABA_B_ receptors to compensate for dysfunction of GABA_A_ receptor-mediated inhibition is transient, as RDD is diminished after 8 weeks of diabetes. Despite this, GABA_B_ receptors remain functional in 8-week diabetic rats, as administration of bicuculline restores RDD in a GABA_B_ receptor-dependent fashion and spinal delivery of baclofen alleviated allodynia. One potential explanation for this progression is that diabetes induces an early disruption of GABA_A_ receptor-mediated spinal inhibition that is initially compensated for by elevated spinal GABA release [20] and GABA_B_ receptor-mediated spinal inhibition, thereby maintaining normal RDD. This compensation is eventually overcome by GABA_A_ receptor dysfunction progressing towards an excitatory phenotype, which manifests as loss of RDD. It remains unclear what changes take place between 4- and 8-week durations of diabetes that promote this shift, as it is unknown whether spinal GABA release remains elevated beyond 4 weeks of diabetes [20]. It has been suggested that presynaptic GABA_B_ receptor function is attenuated as early as 4 weeks after induction of diabetes [42]. However, GABA_B1_ receptor subunit mRNA and protein levels in the dorsal horn also progressively decrease as the duration of experimental diabetes increases [43] and could contribute to a physiological failure of RDD by week 8 while allowing responses to pharmacological manipulation.

RDD has been used to identify the presence of spinal disinhibition in neuropathic pain states in rats and humans [6,32] and may serve as a tool with which to identify pharmacological manipulations that might show efficacy against neuropathic pain. After identifying a role for inhibitory GABA_B_ receptors in normal RDD that remained functional during diabetes, we investigated the efficacy of the GABA_B_ receptor agonist baclofen against indices of neuropathic pain in 4- and 8-week diabetic rats, to determine whether increasing inhibitory drive through this receptor could serve as a potential therapy for painful diabetic neuropathy. Intrathecal baclofen alleviated tactile allodynia in both 4- and 8-week diabetic rats, indicating that GABA_B_ receptor inhibitory function is as relevant to the behavioral manifestations of neuropathic pain in diabetic rats as it is to RDD. The effect of baclofen on RDD could not be evaluated directly because administration of this receptor agonist alters the basal amplitude of the H-reflex [13], which can itself affect the magnitude of RDD [44]. However, these data are consistent with previous demonstrations that systemic and intrathecal baclofen can alleviate mechanical hyperalgesia in diabetic rats [45,46,47,48]. Given that indices of neuropathic pain are alleviated in diabetic rodents by both the GABA_B_ agonist baclofen and the GABA_A_ antagonist bicuculline [7], a combination of these drugs may be an effective means of manipulating spinal GABAergic systems towards pain relief under diabetic conditions.

The carbonic anhydrase inhibitor acetazolamide has been proposed as a viable therapy for treatment of neuropathic pain conditions with underlying spinal disinhibition arising from inversion of the chloride reversal potential [23,29,30,31]. Blocking bicarbonate efflux counteracts disinhibition due to reduced KCC2 and subsequent dysregulation of intracellular chloride, but is not expected to affect normal spinal inhibition [30]. To our knowledge, efficacy against indices of neuropathic pain in diabetes has not been studied but may be predicted by the involvement of decreased spinal KCC2 and disrupted GABAergic inhibitory systems. Spinal delivery of acetazolamide both restored RDD and reversed tactile allodynia in diabetic rats, supporting the concept that RDD can be used to identify drugs that might be of clinical use for the treatment of neuropathic pain conditions arising from spinal disinhibition. Furthermore, the restoration of RDD was dependent upon GABA_A_ receptors, suggesting that the mechanism of action was via restoration of GABA_A_ receptor-mediated inhibitory function. Recent studies have shown that carbonic anhydrase inhibitors also act against indices of pain caused by the chemotherapeutic agent oxaliplatin [49,50,51,52], although in this case modulation of primary afferent TRPA1 and TRPV1 channels secondary to altered cytoplasmic pH has been implicated rather than modulation of spinal inhibitory systems [53]. The utility using RDD as a tool to identify spinally acting analgesics is not limited to painful diabetic neuropathy, as impaired RDD following paw formalin injection was restored by a specific GABAα5 receptor antagonist that concurrently alleviated the accompanying secondary allodynia and hyperalgesic pain behaviors [32].

Our data establish the involvement of both GABA_A_ and GABA_B_ receptors in RDD of the H-reflex. Because impaired RDD has been associated with neuropathic pain arising from spinal disinhibition secondary to reduced spinal KCC2 expression [6,7]. Understanding the underlying receptor systems that contribute to impaired RDD may focus development of novel therapeutic avenues against spinally mediated neuropathic pain. RDD also has the potential to be used to identify specific patients in whom neuropathic pain may have a spinal component, although one caveat is that humans with advanced neuropathy may have diminished or absent H-waves due to myelinated fiber loss—a situation that does not occur in short-term diabetic rodents. While the present studies were performed in a model of type 1 diabetes, impaired RDD has also been noted in type 2 diabetic rats and in humans with type 1 diabetes and painful neuropathy [9]. Moreover, normal rats exhibit reduced RDD following secondary formalin hyperalgesia [32] so that this approach to identifying pain generator sites and personalizing therapy accordingly may be applicable to neuropathic pain of diverse origins. A particularly compelling future vision would be to combine a diagnosis of irritable nociceptor phenotype for identification of peripherally driven pain [3,4] with an evaluation of RDD impairment for the identification of spinal disinhibition in order to select the most rational drug choice for individual patients.

## Data Availability

The data presented in this study are available on reasonable request from the corresponding author.
